# The accuracy of cardiac MRI in differentiating between intra cardiac tumors and thrombi

**DOI:** 10.1186/1532-429X-17-S1-Q70

**Published:** 2015-02-03

**Authors:** Orly Goitein, Einat Slonimasky, Ashraf Hamdan, Yishay Salem, Elio Di Segni, Osnat Konen, Eli Konen

**Affiliations:** 1 Diagnostic Imaging, Sheba Medical Center, Tel Hashomer, Israel; 2 Heart Center, Sheba Medical Center, Tel Hashomer, Israel; 3 Diagnostic Imaging, Schneider Children's Medical Center of Israel, Petach Tiquva, Israel

## Background

Primary or metastatic cardiac tumors are rare.

Echocardiography is limited in differentiating different cardiac masses in particular tumors from thrombi. MRI offers incremental value; optimal tissue differentiation especially with Gadolinium contrast enhancement. The ability to differntiate a cardiac tumor from a cardiac thrombus is crucial for patient management. The purposse of this study was to evaluate the accuracy of CMR in differentiating between intra cardiac tumors and thrombi.

## Methods

Retrospective analysis of a prospectively maintained database (2004-2013) was performed. This included all patients referred for the evaluation of intra-cardiac tumors versus thrombi. CMR sequences included: T2, gradient echo,T1 before and after Gadolinium (Gd) administration, first pass perfusion and delayed enhancement. CMR findings were compared to the definitive diagnosis of the intra cardiac - mass established either by pathology and /or clinical and echocardiographic follow up, when available. Accuracy, sensitivity and positive predictive volume (PPV) of CMR for differentiating between tumor and thrombus were calculated, accordingly.

## Results

Of the 150 patients referred for CMR, intra cardiac masses were detected at in 111 patients. At CMR cardiac tumors were diagnosed in 72 patients, thrombi in 28 patients ; 11 masses were inconclusive. Definitive diagnosis was available in 66 patients including 49 tumors and 17 thrombi. CMR correctly diagnosed 48 out of 49 tumors ; sensitivity PPV and NPV were 97%,97% and 92%, respectively. Thrombi were correctly diagnosed at CMR in 14 out of 17 scans; sensitivity PPV and NPV were 82%,93% and 80%, respectively. The overall accuracy in differentiating intra-cardiac tumors from thrombi was 94%. Of the 11 inconclusive lesions detected, 5 were smaller than 1 cm.

## Conclusions

The different CMR sequences allows reliable tissue characterization, thus enabling an accurate differentiation between intra cardiac tumors and thrombi. This high diagnostic potential is limited in lesion smaller than 1 cm.

## Funding

No external funding was used for this study.

